# Circulating levels and characterization of microparticles in patients with different degrees of glucose tolerance

**DOI:** 10.1186/s12933-017-0600-0

**Published:** 2017-09-19

**Authors:** Alessandra Giannella, Claudia Maria Radu, Lorenzo Franco, Elena Campello, Paolo Simioni, Angelo Avogaro, Saula Vigili de Kreutzenberg, Giulio Ceolotto

**Affiliations:** 1 Department of Medicine-DIMED, University of Padova, Padua, Italy; 2 Department of Chemical Sciences, University of Padova, Padua, Italy

**Keywords:** Microparticles, microRNA, Prediabetes, Type 2 diabetes

## Abstract

**Background:**

Microparticles (MPs) are vesicular structures shed from endothelial or circulating blood cells, after activation or apoptosis, and can be considered markers of vascular damage. We aimed to determine the levels of circulating MPs, their content of miRNA-126-3p and 5p, and their relationship with early endothelial activation/damage, in patients with different degree of glucose tolerance.

**Methods:**

CD62E^+^, CD62P^+^, CD142^+^, CD45^+^ circulating MPs, their apoptotic (AnnexinV^+^) fractions, and miRNA-126 expression were determined in 39 prediabetic (PreDM), 68 type 2 diabetic (T2DM), and 53 control (NGT) subjects, along with main anthropometric and biochemical measurements. MPs were analysed by flow cytometry. miRNA-126 was measured by quantitative real-time PCR. Plasma antioxidant capacity was determined by electronic spin resonance; ICAM-1, and VCAM-1 by ELISA.

**Results:**

Activated endothelial cell-derived MPs (CD62E^+^) were significantly increased in PreDM and T2DM in comparison to NGT (p < 0.0001). AnnexinV^+^/CD62E^+^ MPs and Annexin V^+^ MPs were significantly increased in T2DM compared to PreDM and NGT (p < 0.001); other MPs were not significantly different among groups. Plasma antioxidant capacity was significantly decreased in PreDM and T2DM compared to NGT (p = 0.001); VCAM-1 significantly increased in PreDM and T2DM in comparison to NGT (p = 0.001). miR-126-3p expression, but not miR-126-5p, in MPs, decreased significantly and progressively from NGT, to PreDM, and T2DM (p < 0.001). In PreDM and T2DM, CD62E^+^ MPs level was significantly and negatively associated with plasma glucose (p = 0.004).

**Conclusion:**

We show for the first time that circulating CD62E^+^ MPs level and miR-126-3p content in MPs are abnormal in subjects with pre-diabetes; the content of miR-126-3p correlates with markers of endothelial inflammation, such as VCAM-1, plasma antioxidant capacity, and microparticles, well-accepted markers of endothelial dysfunction.

## Background

Microparticles (MPs) are a heterogeneous population of vesicles, with a diameter of 100–1000 nm, released from the plasma membrane surface of various cell types, such as platelets, erythrocytes, granulocytes, monocytes, lymphocytes, dendritic, and endothelial cells. MPs express proteins specific to the cells they are derived from, thus allowing cell type identification [1].

The release of MPs is a highly controlled process driven by different stimuli such as proinflammatory cytokines, infectious agents, lipoproteins, oxidative stress, or shear stress levels [2, 3]. MPs are also detectable in the peripheral blood of healthy individuals, but they can markedly increase in pathological condition, such as atherosclerosis, and hypertension [4, 5], and can therefore be considered biomarkers of diseases. Furthermore, MPs are capable of directly stimulating intracellular signalling and eliciting cellular responses: several studies have demonstrated the ability of MPs to promote vascular inflammation, and to interfere with coagulation pathway [6, 7].

Diamant and colleagues reported that MPs number and origin were comparable between healthy subjects and well-controlled type 2 diabetic patients; however, the latter group had significantly higher tissue factor-positive MPs [8]. In the Framingham Heart Study, circulating MPs levels were associated with the presence of cardio-metabolic risk factors, particularly dyslipidemia. Intriguingly, in this study, MPs level was not significantly associated with overt diabetes, but the association between triglycerides and circulating MPs was more pronounced in diabetic patients compared to non-diabetic subjects [9].

Recently, it has been demonstrated that, in vitro, elevated glucose induces a marked release of MPs with a distinct molecular composition by endothelial cells with respect to normal glucose treatment [10]. However, the effect, in vivo, of different condition in carbohydrate metabolism on the release of MPs and on the endothelial activation or dysfunction has not been completely investigated.

MPs are one of the major transport vehicles of microRNA (miRNAs), and contribute to the transfer of microRNAs from the parent cell to various target cells by direct cell-to-cell interaction, modulating the expression of specific genes; miRNAs contained in MPs may in turn influence vascular homeostasis [11–13].

Endothelium-derived MPs promote vascular endothelial repair by delivering miR-126-3p into recipient cells; however, MPs obtained in hyperglycaemic conditions show reduced regenerative capacity both in vivo, and in vitro [14]. A reduced expression of miRNA-126 has been shown in susceptible individuals, and in patients with type 2 diabetes mellitus [15, 16]; this suggests that the vascular protection of miRNA-126 is lost in the early phases of glucose intolerance.

The precursor miRNA pre-miR-126 gives rise to two mature strands, miR-126-3p and miR-126-5p, which are the most abundant miRNA in endothelial cells [17]. Deletion of gene encoding pre-miR-126 induces the loss of vascular integrity, and impairment of ischemic neovascularization [18]. The silencing of miR-126-3p induces endothelial inflammatory activation, in vitro [19]; furthermore, transfer of miR-126-3p by MPs released from apoptotic endothelial cells limits the atherosclerotic process, indicating an essential role of miRNA-126 in the endothelial stress response [20, 21].

Glucose metabolism abnormalities are strongly associated with the development of endothelial dysfunction and atherosclerosis [22, 23]. Alterations in carbohydrate metabolism form a continuum, which progressively worsens the cardiovascular health. Early hyperglycemia through a “metabolic memory” triggers endothelial activation as the initial vascular abnormality [24]. In this context, MPs release induced by early hyperglycemia may be considered novel biomarkers of endothelial activation/dysfunction.

Several studies reported that miRNAs represent a very promising tool for diagnosis and prognostic purposes in type 2 diabetes. However, while circulating miRNAs are modulated by a wide range of factors, including sex, age, and drug treatments, miRNAs within MPs can represent a cleaner source of information, mainly due to their nature of communication between tissues, either contained in exosome [25], or extracellular vesicles [24]. Taken together, these findings suggest that circulating MP-packaged miRNAs, in addition to their role as biomarkers, could act as functional mediators in vascular and metabolic diseases.

Therefore, the aim of this study was to determine the level of circulating MPs, related to endothelial activation/dysfunction and to assess the expression of miR-126-3p and miR-126-5p in MPs, across the spectrum of glucose intolerance, in non-diabetic, pre-diabetic and type 2 diabetic patients. We also investigated the relationships among MPs, their miRNA-126 content, metabolic parameters and early endothelial activation markers.

## Materials and methods

### Research design

We identified 160 consecutive subjects, among those attending the outpatient clinic of the Division of Metabolic Diseases of the University of Padua: 53 with normal glucose tolerance (NGT), 39 with prediabetes (PreDM; IFG or IGT defined as impaired fasting glucose or impaired glucose tolerance) and 68 with ascertained type 2 diabetes mellitus (T2DM, >5 years duration). Exclusion criteria were type 1 diabetes, clinically relevant diseases or advanced chronic diabetes complications. NGT and PreDM were assessed by an oral glucose (75 gr) tolerance test, with frequent sampling (−10′, 0′, 20′, 30′, 60′, 90′, 120′, 150′, 180′) for the determination of plasma glucose, C-peptide and insulin concentrations to evaluate, in addition to the glucose metabolism status, the insulin sensitivity index (Si) [26], and the HOMA index [27]. PreDM was defined as IFG [fasting plasma glucose 100–125 mg/dl (5.5–6.9 mmol/l] and/or IGT [2-h plasma glucose 140–199 mg/dl (7.7–11.1 mmol/l)] according to ADA criteria.

Anthropometric and blood pressure measurements were performed in all the subjects and a blood sample in fasting condition was obtained for the determination of glucose, lipid profile, ICAM-1, VCAM-1, plasma antioxidant capacity and MPs.

This study was carried out in accordance with the International Ethical Guidelines and the principles of the Declaration of Helsinki, and was approved by the local Institutional Review Board of the University of Padua Medical Centre. Patients filled out a complete lifestyle questionnaire regarding medical history, current therapy, smoking habits, and physical activity. All subjects signed the informed consent.

### Metabolic parameters, adhesion molecule determinations and antioxidant capacity of plasma

Plasma glucose, insulin, C-peptide, and lipids were determined by enzymatic methods. Plasma levels of soluble adhesion molecules, Intercellular adhesion molecule-1 (ICAM-1), and Vascular cell adhesion molecule (VCAM-1) were measured as markers of early endothelial dysfunction by using a high-sensitivity ELISA assay, according to the manufacturer’s instructions (BioVision, CA, USA). Results were expressed in ng/ml. The intra and inter-assay coefficient of variation were below 10%. All samples were coded for a blinded analysis, and each plasma sample was determined in duplicate.

The antioxidant capacity (AOC) of plasma was evaluated with Electron Spin Resonance (ESR) spectroscopy, by measuring the ESR intensity decay of the nitroxide probe 4-hydroxy-2,2,6,6-tetramethyl-piperidine-1-oxyl TEMPOL (Aldrich, USA), added to the plasma (0.25 mM). The samples were mixed with TEMPOL in quartz capillaries and rapidly inserted into the cavity of a Bruker ER200 ESR spectrometer at room temperature. ESR spectra of the nitroxide probe were recorded at 1 min interval for the first 15 min with the following experimental parameters: magnetic field sweep 60 G, microwave power 1 mW, modulation amplitude 1 G, scan time 40 s as previously described [28]. The initial ESR intensity decay rate is proportional to the antioxidant level. To convert the decay rate into an ascorbate equivalent concentration, we prepared a calibration curve with ascorbate-TEMPOL samples at known ascorbate concentration, to give antioxidant levels [29].

### Microparticles assessment and characterization

Platelet-poor plasma (PPP) was prepared within 3 h of blood collection by double centrifugation (3000*g* for 15 min). 1 ml of PPP was centrifuged at 18,000*g* for 40 min at 4 °C to obtain microparticles (MPs). MPs were resuspended in 200 μl of phosphate-buffered saline (PBS, Sigma, USA) and stored at −80 °C until use. Samples, analyzed only after a single freeze–thaw cycle, were thawed by incubation for 5 min in a water bath at 37 °C immediately before assay.

All assays were performed on a Cytomics FC500 flow cytometer (Beckman Coulter, Miami Florida), as previously described [30]. The MPs gate was established using a blend of mono-dispersed fluorescent beads of three diameters (0.5, 0.9, and 3 μm) (Megamix, BioCytex, DiagnosticaStago, France) [31]. Twenty microliters (µl) of freshly thawed MPs were directly incubated for 15 min at room temperature in the dark with 2 µl of fluorescent-conjugated monoclonal antibodies against cell-type specific antigens and 2 µl of annexinV-FITC (fluorescein isothiocyanate) (Bender MedSystems GmbH, Vienna, Austria). Endothelial-derived MPs were identified usi ng CD62E-PE (phycoerythrin) and platelet-derived MPs using CD62P-PC5 (phycoerythrin-cyanin 5.1) (Beckman Coulter, Miami, Florida); leukocyte-derived MPs using CD45-PC5 (BioLegend Europe, The Netherlands) and Tissue Factor-bearing (TF + MP) with CD142-PE, (clone HTF-1, BD, Biosciences, Milan, Italy). The isotype controls used were IgG1-PC5, clone MOPC-21 (BioLegend Europe), IgG1-PE, clone MOPC-21 (BD Biosciences, Milan, Italy); mouse IgG1-FITC, clone MOPC-21 (BioLegend Europe). The samples were diluted in 500 μl of 0.22 µl filtered Annexin-V kit binding buffer (Bender MedSystems GmbH, Vienna, Austria) before analysis. A total of 20 μl of counting beads with an established concentration (Flow Count™ Fluorospheres, Beckman Coulter, Miami Florida) were added to each sample in order to calculate MPs as absolute numbers per microliter.

### miRNA-126 expression in microparticles

Microparticles from plasma of all the patients were isolated, as above described. Supernatants were removed, and the pellet composed of MPs was used for the determination of the miR-126-5p and miR-126-3p expression. RNA extraction from MPs was determined using RNeasy MinElute Cleanup kit (Qiagen, Hilden, Germany). cDNA was synthesized using specific miRNA primers (Applied Biosystems, USA) for miR-126-3p and miR-126-5p in TaqMan microRNA Reverse Transcription kit (Applied Biosystems,USA). Expression of miR-126-3p and miR-126-5p were assessed by quantitative real-time RT-PCR (qPCR), using a CFX96 PCR detection system (Biorad CFX 96). The RNU48 snRNA was used as an internal control and to normalize miR-126-5p and miR-126-3p expression (Applied Biosystems, USA) [32].

### Statistical analysis

Continuous variables are expressed as mean ± standard error and categorical variables as percentage. Non-normal variables at the Kolmogorov–Smirnov test were log-transformed before analysis. Comparisons between 2 or more groups were performed with the Student’s t test or ANOVA, respectively for continuous data. The Bonferroni post hoc test was applied. To determine the association between MPs type and studied variables, univariate analyses were first run and then multiple linear regression analysis was run entering MPs as dependent variable, and explanatory covariates chosen among those showing significant (post hoc p < 0.05) associations in univariate group analysis. Statistical significance was accepted at p < 0.05, and SPSS ver. 22 was used.

## Results

### Clinical characteristics and adhesion molecules

Main demographic and clinical characteristics of the study subjects are reported in Table 1. Subjects were divided into three categories according to their degree of glucose tolerance. T2DM and PreDM subjects showed significantly increased BMI, systolic blood pressure values, heart rate, and fasting plasma glucose levels in comparison to controls. PreDM subjects showed significantly lower plasma glucose levels and higher blood pressure values compared to T2DM. PreDM was associated with a worse insulin-resistant state, compared to NGT, as demonstrated by Si (8.2 ± 1.6 vs 16.5 ± 2.2 10^−4^ dl/kg/min µU/ml; p = 0.017) and HOMA index (4.0 ± 0.5 vs 1.2 ± 0.2; p < 0.001) measurements.

**Table 1:** Clinical characteristics of the study cohort

	NGT (n = 53)	PreDM (n = 39)	T2DM (n = 68)	p value
Sex (M/F)	30/23	31/8	42/26	0.124
Age (years)	57 ± 1	60 ± 1	60 ± 1	0.093
BMI (kg/h^2^)	25 ± 0.4	28 ± 0.6^#^	30 ± 1.6^#^	<0.0001
SBP (mmHg)	120 ± 2	143 ± 4^#, ¶¶^	133 ± 2^¶^	<0.0001
DBP (mmHg)	78 ± 1	91 ± 2^#,##^	80 ± 1	<0.0001
HR (bpm)	67 ± 2	74 ± 2^¶^	74 ± 2^¶^	0.032
Plasma glucose (mg/dl)	86 ± 1	106 ± 3^§,##^	160 ± 8^#^	<0.0001
Total cholesterol (mg/dl)	190 ± 3	198 ± 6	188 ± 5	0.507
HDL cholesterol (mg/dl)	51 ± 2	47 ± 2	50 ± 2	0.155
LDL cholesterol (mg/dl)	121 ± 3	123 ± 5	113 ± 5	0.261
Triglyceride (mg/dl)	113 ± 10	147 ± 9^¶^	134 ± 13	0.096

Data presented as Mean ± ES (p value ANOVA and Bonferroni test)

*NGT* glucose normotolerant, *PreDM* pre-diabetic, *T2DM* type 2 diabetic mellitus, *BMI* Body Mass Index, *SBP* systolic blood pressure, *DBP* diastolic blood pressure, *HR* heart rate, *bpm* beats per minute, *HDL* high density lipoprotein, *LDL* low density lipoprotein

^#^p < 0.0001 vs NGT

^§^p < 0.01 vs NGT

^¶^p < 0.05 vs NGT

^##^p < 0.0001 vs T2DM

^¶¶^p < 0.05 vs T2DM

### Microparticles levels

**Fig. 1:**
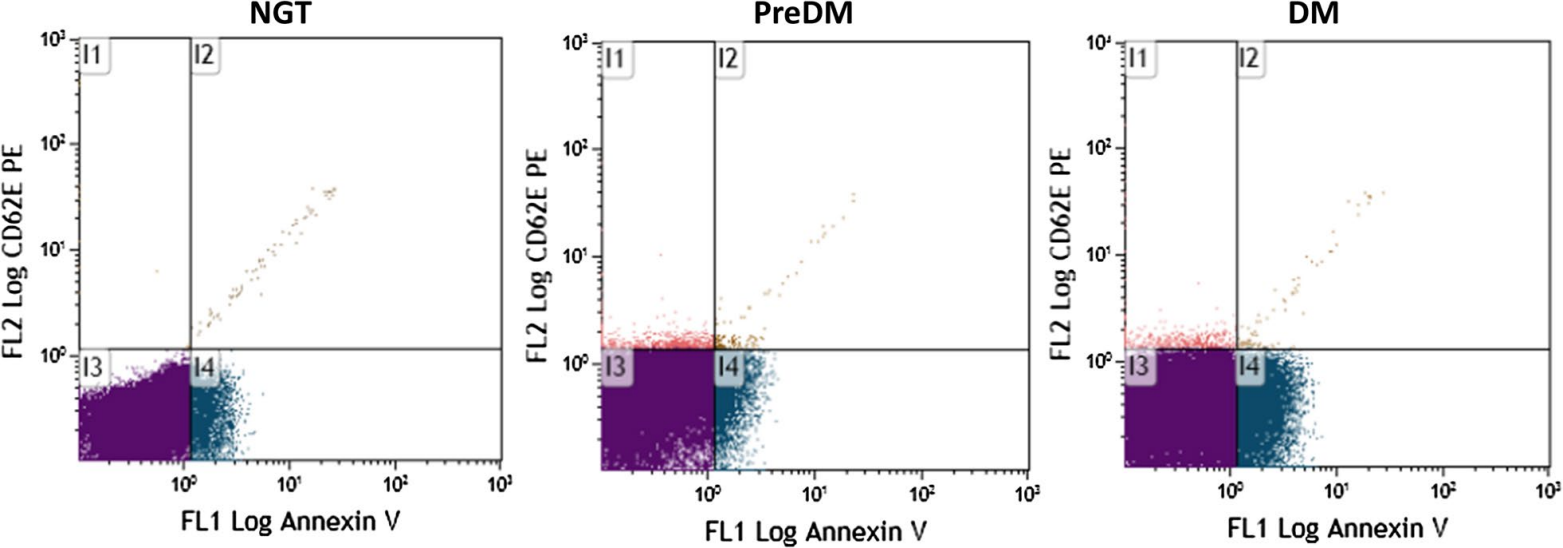
Representative MPs profiles in plasma sample of NGT, PreDM and T2DM, flow cytometric scatter plots. I1 quadrant displays the number of CD62E-positive MPs, I2 displays AnnexinV/CD62E positive MPs, I3 displays AnnexinV/CD62E negative and I4 displays AnnexinV positive MPs

We determined the levels of circulating MPs obtained from activated endothelial cells (CD62E^+^), tissue factor-bearing MPs (CD142^+^), leukocyte-derived MPs (CD45^+^), and activated platelet-derived (CD62P^+^) MPs according to degree of glucose tolerance (Table 2). The concentration of circulating CD62E^+^ MPs was significantly increased in PreDM and in T2DM compared to NGT subjects. T2DM patients showed significantly higher values of AnnexinV^+^/CD62E^+^ and AnnexinV^+^ MPs compared to NGT and PreDM. No other significant differences were observed for all the other kinds of MPs investigated. Figure 1 shows a representative scatter plot from cytometer of circulating CD62E^+^ MPs and the apoptotic fraction from NGT, PreDM and T2DM. PreDM and T2DM exhibited a larger population of MPs in quadrant I1 (CD62E^+^ MPs) than NGT, as well as T2DM showed more positive MPs in quadrant I2 and I4 (respectively AnnexinV^+^/CD62E^+^ and AnnexinV^+^ MPs) compared to NGT and PreDM.

**Table 2:** Circulating levels of different MPs subtypes in the study cohort

Origin	NGT (n = 53)	PreDM (n = 39)	T2DM (n = 68)	p value
AnnexinV^+^	3445 ± 500	4385 ± 800	14,991 ± 1700^#^	<0.001
Endothelial cells				
CD62E^+^	168 ± 48 ± 1	948 ± 118^##^	693 ± 81^##^	<0.0001
AnnexinV^+^ CD62E^+^	65 ± 6	66 ± 10	100 ± 9^#^	<0.0001
Tissue factor				
CD142^+^	3.57 ± 0.39	2.67 ± 0.28	3.41 ± 0.32	0.127
AnnexinV^+^ CD142^+^	37.14 ± 2.76	32.94 ± 2.82	29.88 ± 1.77	0.146
Leukocytes				
CD45^+^	277 ± 20	296 ± 17	308 ± 15	0.476
AnnexinV^+^ CD45^+^	48.47 ± 5.06	35.23 ± 4.65	38.28 ± 3.07	0.121
Platelets				
CD62P^+^	220 ± 48	207 ± 74	135 ± 21	0.458
AnnexinV^+^ CD62P^+^	124 ± 56	88 ± 28	113 ± 14	0.505

Data presented as Mean ± ES (p value ANOVA and Bonferroni test)

*NGT* glucose normotolerant, *PreDM* pre-diabetic, *T2DM* type 2 diabetic mellitus

^#^p < 0.0001 vs NGT and PreDM

^##^p < 0.0001 vs NGT

### Correlations between CD62E^+^ MPs and other variables

We then investigated possible correlations between the levels of CD62E^+^ MPs and the other studied parameters. Positive correlations were observed between CD62E^+^ MPs and plasma glucose (r = 0.23; p = 0.005), BMI (r = 0.21; p = 0.012), systolic blood pressure (r = 0.17; p = 0.042) and diastolic blood pressure (r = 0.16; p = 0.048).

At multiple linear regression analysis, entering CD62E^+^ MPs as dependent variable, and systolic blood pressure (Beta = 0.9; p = 0.32), diastolic blood pressure (Beta = 0.14; p = 0.10), BMI (Beta = 0.12; p = 0.2), and plasma glucose (Beta = 0,19; r = 0.037) as independent variables, plasma glucose level was the strongest independent predictor of CD62E^+^ MPs. AnnexinV^+^ MPs levels were positively associated with plasma glucose (r = 0.27; p = 0.001) while AnnexinV^+^/CD62E^+^ MPs were not significant correlated with clinical parameters.

### Plasma antioxidant level and early adhesion markers

**Fig. 2:**
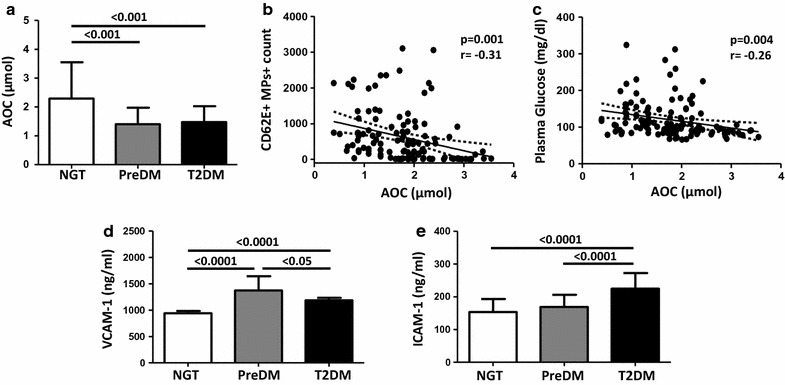
Plasma Antioxidant level, ICAM-1 and VCAM-1 measurements in NGT, in PreDM, and in T2DM. **a** Plasma antioxidant capacity (AOC) measured with ESR spectroscopy. **b**, **c** Correlations between AOC and CD62E + MPs, AOC and plasma glucose, respectively. **d**,** e** VCAM-1 and ICAM-1 plasma levels determined by ELISA, respectively. Data are expressed as Mean ± DS

The antioxidant level in plasma from all the subjects was assessed by the measurement of the ascorbate-equivalent concentration with ESR spectroscopy. As shown in Fig. 2a, a large decrease of the antioxidant level was found in plasma from PreDM, and T2DM in comparison to NGT (1.4 ± 0.1 and 1.5 ± 0.07 vs 2.3 ± 0.1; p = 0.001). Plasma antioxidant level was negatively correlated with CD62E + MPs (r = −0.31; p = 0.001) and with plasma glucose (r = −0.26; p = 0.004) (Fig. 2b, c).

Then, in all the subjects, we also measured the amount of plasma VCAM-1 and ICAM-1, as early endothelial activation markers. VCAM-1 levels showed a significant increase in PreDM and T2DM in comparison to NGT, while ICAM-1 level was significantly increased in T2DM in comparison to NGT and PreDM subjects (Fig. 2d, e).

### miRNA-126 expression in microparticles

**Fig. 3:**
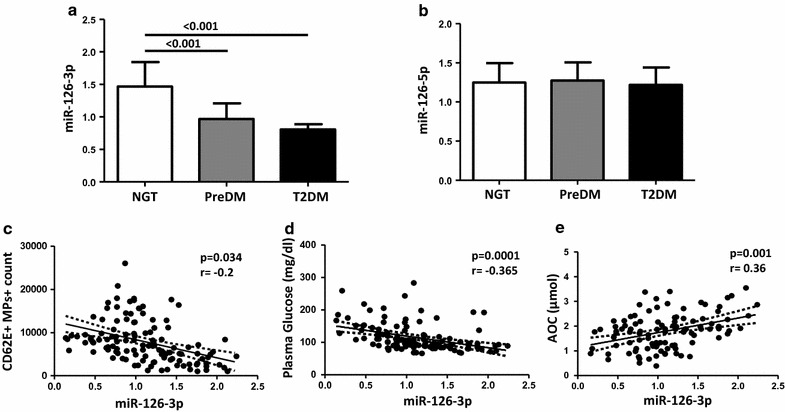
miR-126-3p and miR-126-5p expression from circulating MPs of NGT, PreDM and T2DM patients. **a**, **b** miR-126-3p and miR-126-5p expression determined by qPCR, respectively. Data are expressed as Mean ± DS. **c**–**e** Correlations between expression of miR-126-3p and the number of circulating CD62E^+^ MPs, plasma glucose levels and plasma antioxidant capacity (AOC) respectively

Since MPs are the major carriers of miRNAs in circulation, we analysed the expression of miRNA-126, i.e. of its two strands miR-126-3p and miR-126-5p in MPs isolated from the plasma of PreDM, T2DM and control subjects. The level of miR-126-3p was significantly higher in control compared to PreDM (p < 0.001 after Bonferroni correction) and T2DM (p < 0.001) subjects, while it was comparable between PreDM and T2DM patients (Fig. 3a). On the other hand, we did not observe any significant difference for miR-126-5p concentrations among all the three groups (Fig. 3b).

### Correlations between miRNA-126 expression and other variables

We investigated the correlations between miR-126-3p and miR-126-5p and all studied parameters, in all the subjects. miR-126-3p was significantly and negatively associated with CD62E^+^ MPs (r = −0.2; p = 0.034) (Fig. 3c), with plasma glucose (r = −0.365; p = 0.0001) (Fig. 3d), VCAM-1 (r = −0.25; p = 0.010) and positively with antioxidant plasma level (r = 0.36; p = 0.001) (Fig. 3e).

At multiple linear regression analysis, entering miR-126-3p as dependent variable, and the variables significantly associated with miR-126-3p at simple regression analysis as independent ones, only CD62E^+^ MPs (Beta = −0.300; p = 0.003), and fasting plasma glucose (Beta = −0.286; p = 0.003) remained significant predictors of reduced levels of MPs miR-126-3p.

On the other hand, we did not find any significant correlation with miR-126-5p.

## Discussion

The present study was designed to characterize and to define the role of several circulating levels of MPs involved in endothelial activation/dysfunction in subjects with different degrees of abnormal glucose metabolism. To this purpose, we determined: (a) CD62E^+^ (E-selectin) MPs that are released by the activated endothelium [33, 34]; (b) CD62P^+^ (P-selectin) MPs that are mainly shed by platelets in response to cell activation, and are involved in inflammation and thrombosis [7, 35, 36]; (c) CD45^+^ MPs that derive from lymphocytes, and have been associated with the type of subclinical atherosclerotic lesions [37]; and (d) CD142^+^ (tissue factor) MPs which expression is induced on the surface of damaged or activated endothelial cells [38].

### MPs pattern in different degrees of glucose tolerance

We demonstrated that only circulating CD62E^+^ MPs levels were significantly increased in T2DM and in PreDM compared to NGT subjects, while the levels of AnnexinV^+^/CD62E^+^ MPs and AnnexinV^+^ MPs were higher in T2DM, but not in PreDM, suggesting only in these patients an increased apoptosis. This indicates not only that CD62E^+^ MPs can be considered an early marker of endothelial dysfunction in hyperglycaemic patients, but also that an early activated endothelial state is already present in the prediabetic condition.

The association between CD62E^+^ MPs and reduced antioxidant capacity and increased production of adhesion molecules, both established signs of endothelial dysfunction, strengthen their (pre)atherosclerotic role. The observed strong positive correlation between CD62E^+^ MPs and plasma glucose confirms the relationship between hyperglycemia and endothelium dysfunction. Furthermore, multivariable analyses indicate that hyperglycemia is the primary metabolic defect associated with the release of CD62E^+^ MPs as the correlation persisted after adjusting for BMI and blood pressure. Finally, we show that the content of miR-126-3p was markedly reduced in MPs from PreDM and T2DM in comparison to NGT, and negatively correlated with plasma glucose. These findings support the hypothesis of an important role of MPs as carriers of miRNAs, in the regulation of vascular health, which is altered by hyperglycemia, and in particular in diabetic state. To our knowledge, this is the first study reporting a detailed analysis of metabolic parameters in relation to circulating MPs levels, their miRNA content, and to markers of endothelial dysfunction, in a cohort of non-diabetic, pre-diabetic and diabetic patients.

The abnormal release of CD62E^+^ MPs in PreDM could represent a marker of an altered endothelial phenotype that may progressively result to an increase of the apoptotic fraction of MPs levels, as observed in type 2 diabetic patients. Therefore, while pre-diabetes and type 2 diabetes can be seen as a metabolic continuum, the persistence of hyperglycemia (T2DM) appears to be associated with a different pattern of circulating MPs, characterized by the presence of an increased apoptosis, which may reflect a change from an endothelial activation to dysfunction. Our findings back those of Tramontano et al. who showed a marked increase of apoptotic fraction of circulating endothelial MPs from T2DM in comparison to NGT [39].

### MPs and markers of endothelial inflammation

We found that circulating CD62E^+^ MPs are inversely associated with the plasma antioxidant capacity, while there is a direct correlation with the level of VCAM-1; this suggests that the reduction of plasma antioxidant capacity may promote the release of VCAM-1 through circulating CD62E^+^ MPs signalling. In our study, VCAM-1 levels showed a significant increase in PreDM, and T2DM, although the significance of VCAM-1 levels in prediction of T2DM should be improved by the addition of other inflammatory markers, such as E-selectin [40]. Overall these findings support the hypothesis that the excess of CD62E^+^ MPs release from endothelial cells is an early abnormality of vascular disease as it can be observed in prediabetic subjects who show already higher CD62E^+^ MPs levels than controls.

### miR-126-3p content in MPs from patients with different degrees of glucose tolerance

Several studies have described MPs as “cargos” of information in blood vessel wall under pathological situations such as hypertension, myocardial infarction, and metabolic syndrome [4]. In this context, we observe that the expression of miR-126-3p in MPs reduces progressively from PreDM to T2DM, while miR-126-5p content is similar to control subjects. miR-126-3p levels exert protective vascular effects promoting vascular endothelial growth factor (VEGF) signalling [41], and through other protective mechanisms [15]. Our results therefore provide new evidence for the existence of a link between MPs release and miRNA-126 content in hyperglycaemic conditions. Moreover, we found for the first time an inverse correlation between miR-126-3p content in MPs and fasting glucose level, indicating a strict glycemia-sensitivity of this biomarker. In accordance with our clinical data, Jansen et al. demonstrated that MP-bound miR-126 was significantly reduced in diabetic patients, and endothelial cell-derived MPs were shown to be the major source of miR-126-containing MPs. They also verified that miR-126 content in endothelial cell-derived MPs was significantly reduced in high glucose condition, in vitro, strengthening our findings [42]. Although we can not provide any direct evidence of MPs in vascular repair capacity, the negative associations between miR-126-3p and CD62E^+^ MPs, and between miR-126-3p and plasma VCAM-1 levels suggest a possible role of endothelial MPs as modulators of endothelial activation/dysfunction, through their miR-126-3p content. Moreover, we have also found a positive correlation between miR-126-3p and the plasma antioxidant capacity, confirming the presence of an association between a pro-oxidant and inflammatory imbalance and abnormal circulating CD62E^+^ MPs.

Mechanistically, we still do not know whether an increase of CD62E^+^ MPs from endothelial cells causes endothelial dysfunction per se or whether it represents simply a marker of endothelial activation/dysfunction in patients with altered glucose metabolism. However, several studies suggest a causal effect of hyperglycemia in altering the physical composition and molecular assessment of endothelial MPs, increasing their capacity for interaction with the vasculature, for the induction of coagulation, and oxidative stress, thus impairing vascular reactivity [10, 43, 44].

### Limitations

This study has limitations: its cross-sectional nature does not allow to draw definite conclusions on MPs evolution at the single-patient level. The sample size is relatively small, but analyses of the levels of MPs are quite robust and reach statistical significance. The loss of miR-126 expression in plasma of diabetic patients has been extensively reproduced, therefore, we did not perform the same analysis in total plasma, to confirm this datum in our samples. On the other hand, a comparative study could strengthen our hypothesis, showing that this trend is mainly attributable to microparticles. Moreover, the sorting of different pattern of subpopulations of MPs may be useful in future research to understand the different epigenetic information present in MPs, particularly miRNAs. Notwithstanding these limitations, our data may have pathophysiological implications: they demonstrate that MPs expression of endothelial activation may represent a reliable tool for early detection of vascular dysfunction in prevention screening.

## Conclusion

We show for the first time that circulating CD62E^+^ MPs level and miR-126-3p content in MPs are abnormal in subjects with different degrees of glucose tolerance; miR-126-3p correlates with markers of endothelial inflammation, such as VCAM-1, plasma antioxidant capacity, and microparticles, well-accepted markers of endothelial dysfunction.

## Abbreviations

MPs: microparticles
miRNA: microRNA
NGT: glucose normotolerant
PreDM: pre-diabetes
T2DM: type 2 diabetes mellitus
ADA: American Diabetes Association
BMI: Body Mass Index
SBP: systolic blood pressure
DBP: diastolic blood pressure
HR: heart rate
bpm: beats per minute
HDL: high density lipoprotein
LDL: low density lipoprotein
ICAM-1: intercellular adhesion molecule-1
VCAM-1: vascular cell adhesion molecule-1
AOC: plasma antioxidant capacity
ESR: electron spin resonance
TEMPOL: 4-hydroxy-2,2,6,6-tetramethyl-piperidine-1-oxyl
PPP: platelet-poor plasma
PBS: phosphate-buffered saline
ES: error standard
ANOVA: analysis of variance
VEGF: vascular endothelial growth factor
